# The Effect of Training on the Expression of Protein and Metabolites in the Plasma Exosomes of the *Yili* Horse

**DOI:** 10.3390/ani16020158

**Published:** 2026-01-06

**Authors:** Xinxin Yuan, Xinkui Yao, Yaqi Zeng, Jianwen Wang, Wanlu Ren, Tongliang Wang, Xueyan Li, Lipin Yang, Xixi Yang, Jun Meng

**Affiliations:** 1 College of Animal Science, Xinjiang Agricultural University, Urumqi 830052, China; 2 Institute of Animal Science, Xinjiang Academy of Animal Sciences, Urumqi 830000, China; 3 Xinjiang Key Laboratory of Equine Breeding and Exercise Physiology, Urumqi 830052, China

**Keywords:** *Yili* horse, sports training, plasma exosomes, proteomics, metabolomics

## Abstract

Objective: The effects of physical exercise on protein expression and metabolites in the plasma exosomes of *Yili* horses are not yet clear. This study aims to elucidate the effects of exercise training on the expression of plasma extracellular vesicle proteins and metabolites in *Yili* horses through multi-omics analysis, providing reference indicators for the training and performance evaluation of *Yili* horses. Methods: Eight healthy 3-year-old *Yili* horses were selected for the study, with four untrained horses as the control group and four trained horses as the training group. After conducting proteomic and metabolomic analyses of the plasma exosomes of each group of horses, the differences in protein expression and metabolites were analyzed. Results: Compared with the control group, training significantly increased the levels of proteins in the plasma exosomes of *Yili* horses that promote muscle function and repair and regulate metabolism and immune function. In addition, carbohydrates and their metabolites were significantly reduced. Conclusion: Training significantly altered the protein composition and metabolites of *Yili* horse exosomes. These changes mainly provide new insights for the training and evaluation of *Yili* horse exercise ability, but further research is still needed.

## Simple Summary

The effects of training on the expression of extracellular vesicle proteins and metabolites in Yili horse plasma were detected and analyzed through proteomics and metabolomics. The data shows that proteins that promote muscle function and repair, regulate metabolism and immune function in the plasma exosomes of Yili horses significantly increased after training, while metabolites related to carbohydrates and their metabolites significantly decreased. Combined analysis of proteome and metabolome data revealed that training had similar effects on the biological functions of proteins and metabolites in exosomes, indicating that training significantly altered the protein and metabolite composition of Yili horse exosomes.

## 1. Introduction

The horse industry holds significant economic value [1], with horse racing being one of the sectors that demand high athletic performance from horses [2,3,4]. The *Yili* horse, a famous breed for horse racing [5], is a hybrid of the Kazakh horse and the *Orlov Trotter*, *Don*, *Budyonny*, and *Akhal*-*Teke* horse [5]. Sports training can regulate the epigenetics [6], gene expression [7], and metabolism of horses [8], thereby enhancing their athletic abilities [9]. Enhancing athletic ability is beneficial for increasing the value of horses [10]. Currently, the assessment of a horse’s athletic capability is subjectively conducted through experience and professional observation [11]. Finding more objective evaluation standards is of positive significance for optimizing the assessment criteria for equine athletic performance.

Maintaining the athletic performance of horse racing is an important aspect of the horse racing industry [12], and appropriate training can effectively improve and maintain this performance [13]. During exercise, a series of bioactive substances are released into the circulatory system [14], including cell-secreted vesicles with biological activity [15]. Exosomes are one type of these vesicles, containing RNA and proteins [16], and are present in bodily fluids such as plasma [17], breast milk [18], and cerebrospinal fluid [19]. They can promote corneal endothelial regeneration [20], improve bone microstructure and the accumulation of bone marrow fat [21], participate in the development of cardiovascular health and disease [22], and serve as potential biomarkers for disease diagnosis [23,24] as well as for preclinical and clinical assessments [25]. There are abundant exosomes in plasma [26], and it has been found that training has a significant impact on human plasma exosome levels [27]. Our previous research has shown that competition has a significant impact on the composition of *Yili* horse plasma exosomes; however, the impact of training on the plasma exosomes of *Yili* horses is not yet clear.

Exercise can significantly change protein expression in male exosomes [28], and studies on mice have shown that exercise can significantly affect the protein expression of plasma exosomes [29]. Research on horses has indicated that prolonged aerobic exercise induces significant changes in plasma protein modifications [30]. However, the impact of exercise on the protein expression of equine plasma exosomes is still unclear.

The development of metabolomics has made the study of metabolic mechanisms popular in human exercise and has provided important insights into the mechanisms of athletic ability [31]. Various exosomes are hypothesized to transport exercise-related metabolites [32] and may be involved in exercise-induced adaptive intercellular communication [33]. However, the changes in metabolites in equine plasma exosomes due to exercise are not well understood.

Multimodal omics analyses have advanced research related to exercise [34], but the effects of exercise training on the protein expression and metabolomics of *Yili* horse plasma exosomes are still not clear. Through systematic training, the exercise ability of horses can be effectively improved [35], which may be related to changes in plasma exosomes. We hypothesize that exercise training has a significant impact on the plasma exosomes of *Yili* horses. Analyzing the impact of training on plasma exosomes provides a reference for identifying plasma exosome factors that may improve the exercise ability of *Yili* horses, and provides objective indicators for analyzing the exercise capacity of untrained *Yili* horses. This is positively meaningful for improving the breeding work of *Yili* horses and selecting horses with different athletic abilities. In this study, we investigate the differences in protein expression and metabolites in the plasma exosomes of *Yili* horses subjected to exercise training using proteomics and metabolomics approaches. Our goal is to explore the effects of exercise on protein expression and metabolites in *Yili* horse plasma exosomes, aiming to identify potential exosome-related biomarkers that could enhance the athletic performance of *Yili* horses and provide a reference for a training and evaluation system for their athletic capabilities.

## 2. Materials and Methods

### 2.1. Experimental Animals and Grouping

In this study, eight adult male *Yili* horses, each three years old, were selected as research subjects. Four untrained horses served as the control group, while four horses that had completed a year of training formed the training group. Plasma exosomes were extracted after a year of uninterrupted training, with no breaks in their training regimen in the week preceding sampling. The trained horses began their training at two years old and were mature enough to be registered for the 2023 “China Horse Club Yueyang Tower Silk Road Cup” *Yili* Horse Speed Performance Test, covering a distance of 5000 m. These horses were healthy, having successfully passed equine and anti-doping tests. The control group horses had not undergone any formal training and were kept under grazing conditions without engaging in any exercise or physical labor beyond their daily activities in the recent week. All horses involved were medication-free, had no history of illness, and were in good health.

### 2.2. Ethical Statement

All procedures involving animal experiments were established according to Chinese animal welfare legislation, and all animal care and usage procedures adhered to the guidelines set by the Institutional Animal Care and Use Committee of Xinjiang Agricultural University (Approval Number: 2023020).

### 2.3. Blood Collection

Blood samples were drawn from the horses’ jugular veins in a resting state before their daily exercise at 12:00 PM. Each horse contributed a 20 mL blood sample, collected using EDTA-K2 anticoagulant tubes, followed immediately by the extraction of exosomes.

### 2.4. Exosome Extraction

According to previous research [36,37], the steps for extracting extracellular vesicles in this study are as follows. In general, after separating the horse plasma, it was stored in liquid nitrogen. Before extraction, the plasma was thawed at 37 °C. It was then centrifuged at 2000× *g* for 30 min at 4 °C, and the supernatant was transferred to a new centrifuge tube. It was then centrifuged at 10,000× *g* for 45 min at 4 °C to remove larger vesicles. The supernatant was filtered through a 0.45 μm filter, and the filtrate was centrifuged at 100,000× *g* for 70 min at 4 °C. The supernatant was removed, and the pellet was resuspended in 10 mL of pre-cooled 1×PBS. It was then centrifuged again at 100,000× *g* for 70 min at 4 °C. The supernatant was removed, and the pellet was resuspended in 200 μL of pre-cooled 1×PBS, and then stored at −80 °C.

### 2.5. Transmission Electron Microscopy Observation

Observation was conducted using a Hitachi HT-7700 (Hitachi, Tokyo, Japan) model projection electron microscope. In total, 10 μL of exosomes were taken out, and 10 μL of the sample was drawn and dropped onto the copper mesh to precipitate for 1 min. The supernatant was absorbed with filter paper, and 10 μL of uranyl acetate was dropped onto the copper mesh to precipitate for 1 min. After drying at room temperature for a few minutes, electron microscope detection and imaging were carried out at 100 kv to obtain the results of transmission electron microscopy imaging (×20,000).

### 2.6. Quantitative Proteomics

This study employs the diaPASEF acquisition mode of the timsTOF Pro2 series mass spectrometer (Bruker, Ettlingen, Germany) for exosome differential quantitative proteomic analysis. The overall procedure involves adding a lysis buffer (8 M urea) containing 1 mM PMSF and 2 mM EDTA (final concentration) to the sample, followed by incubation for 5 min and ultrasonic lysis for another 5 min. The lysate is then centrifuged at 4 °C, 15,000× *g* for 10 min, and the supernatant is collected. The total protein concentration is determined through BCA protein quantification analysis. Based on the protein concentration, an equal volume of protein solution is taken, and the volume is made up to 200 μL with 8 M urea. Then, it is reduced with 10 mM DTT at 37 °C for 45 min, and alkylated with 50 mM iodoacetamide (IAM) in a dark room at room temperature for 15 min. Four times the volume of pre-cooled acetone is added to the protein solution, and it is precipitated at −20 °C for 2 h. After centrifugation, the protein precipitate is dried and resuspended in 200 μL of 25 mM bicarbonate solution and 3 μL of trypsin (Promega), and digested overnight at 37 °C. After digestion, the peptides of each sample are desalted on a C18 column, concentrated by vacuum centrifugation, and re-dissolved in 0.1% (*v*/*v*) formic acid for machine analysis.

### 2.7. Liquid Chromatography–Tandem Mass Spectrometry (LC-MS/MS)

This study conducts full-spectrum metabolome detection through liquid chromatography–tandem mass spectrometry. The overall procedure is as follows: Exosome samples are taken out from a −80 °C freezer and thawed on ice (all subsequent operations are required to be performed on ice). Then, 500 μL of 80% methanol aqueous internal standard extractant is added, followed by vortexing for 3 min. The centrifuge tube is quickly frozen in liquid nitrogen for 5 min, then thawed on ice for 5 min, followed by another 5 min of thawing on ice and vortexing for 2 min. After repeating the freezing, thawing, and vortexing process three times, the sample is centrifuged at 12,000 r/min for 10 min at 4 °C. The supernatant (450 μL) is transferred to a new centrifuge tube and concentrated until completely dry. Then, it is re-dissolved in 100 μL of 70% methanol water, vortexed for 3 min, and sonicated in an ice-water bath for 10 min. After centrifugation at 12,000 r/min for 3 min at 4 °C, the supernatant (80 μL) is transferred to the corresponding sample vial for machine analysis.

### 2.8. Bioinformatics Analysis and Statistical Analysis

Gene Ontology (GO) analysis using http://geneontology.org/ (accessed on 3 January 2024), Cluster of Orthologous Groups of proteins (COG) analysis using http://www.ncbi.nlm.nih.gov/COG (accessed on 3 January 2024), and Kyoto Encyclopedia of Genes and Genomes (KEGG) analysis using https://www.genome.jp/kegg/ (accessed on 3 January 2024) were conducted. The data are presented as mean values with standard errors. Student’s *t*-test was utilized to evaluate the differences between groups. A *p*-value of less than 0.05 was deemed statistically significant. Fold Change (FC) was used to represent the ratio of protein expression levels between two groups of samples, and the base 2 logarithm (Log, FC) was taken for analysis and visualization. Generally defined as Log, FC ≥ 0.585 (i.e., FC ≥ 1.5 or ≤ 0.6667) indicates significant differences. Statistical analysis was performed using built-in functions or related packages in R language, and graphs such as volcano and heatmaps were drawn using the ggplot2 package (3.3.5) in R software (4.2.0). The Complex Heatmap package (2.12.0) was used to draw clustering heatmaps.

## 3. Results

### 3.1. Extraction and Identification of Exosomes from Yili Horse Plasma

**Figure 1:**
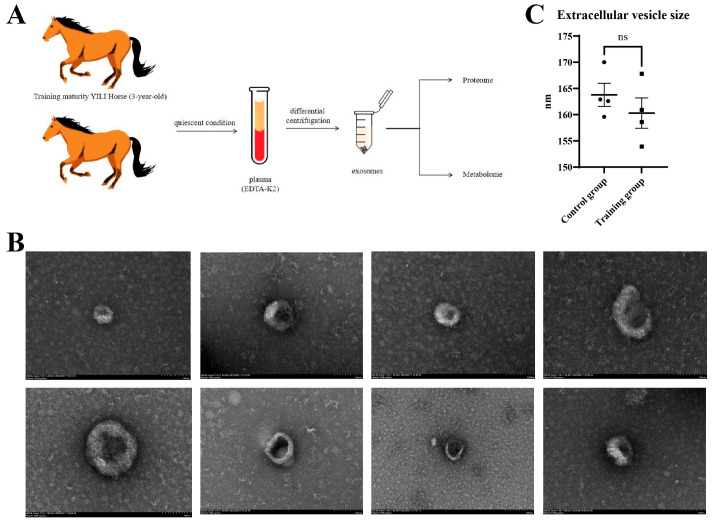
Characteristics of exosomes isolated from *Yili* horse blood. (**A**) Roadmap for this study. (**B**) Representative images of blood-derived exosomes from the (Up) control group and (Down) training group obtained by transmission electron microscopy (Scale bar = 100 nm). (**C**) Comparison of exosome particle size analysis results. Error bar = SEM, ns = not significant.

Figure 1A presents the research pathway employed in this study. After isolating plasma exosomes from both groups of *Yili* horses, we utilized scanning electron microscopy (SEM) for identification purposes, as depicted in Figure 1B. Subsequent analysis of the particle sizes indicated that there was no significant disparity in the exosome sizes between the two groups, as demonstrated in Figure 1C.

### 3.2. Training Significantly Altered Protein Expression of Plasma Exosomes in the Yili Horse

**Figure 2:**
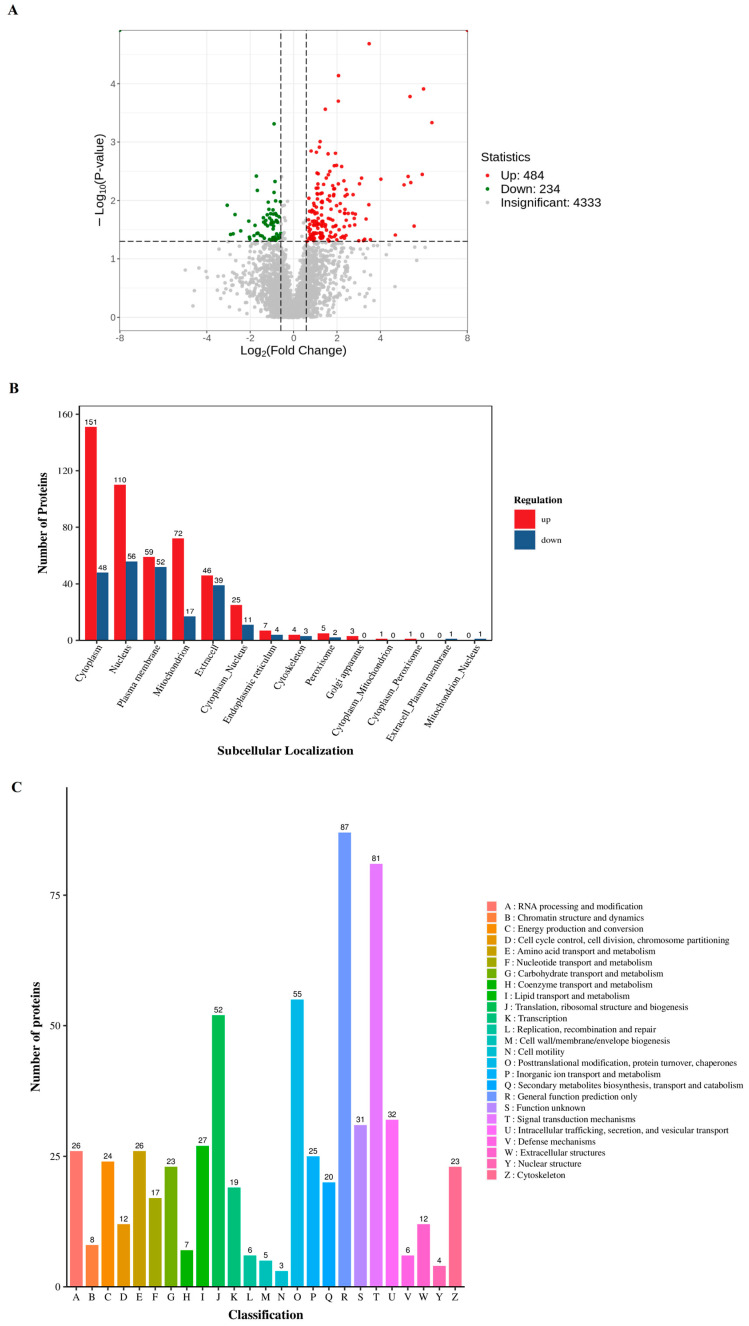
Proteomics to analyze the protein expression within the exosomes. (**A**) Volcano plot of differential exosome proteins. The horizontal axis represents log_2_ of the difference multiple, the vertical axis represents −log_l0_ *p*-value, and red and green dots represent up-regulated and down-regulated differential proteins, respectively. Gray dots represent proteins with no significant differences. (**B**) Comparison bar chart of subcellular localization results of up- and down-regulation. The horizontal axis represents the subcellular location, the vertical axis represents the number of differentially expressed proteins annotated to this subcellular location, and red and blue colors represent up-regulated and down-regulated differentially expressed proteins, respectively. (**C**) Bar chart of Cluster of Orthologous Groups of proteins. The horizontal axis represents KOG functional classification, the vertical axis represents the number of differentially expressed proteins annotated to the corresponding function, and the legend on the right represents the description of functional classification.

**Figure 3:**
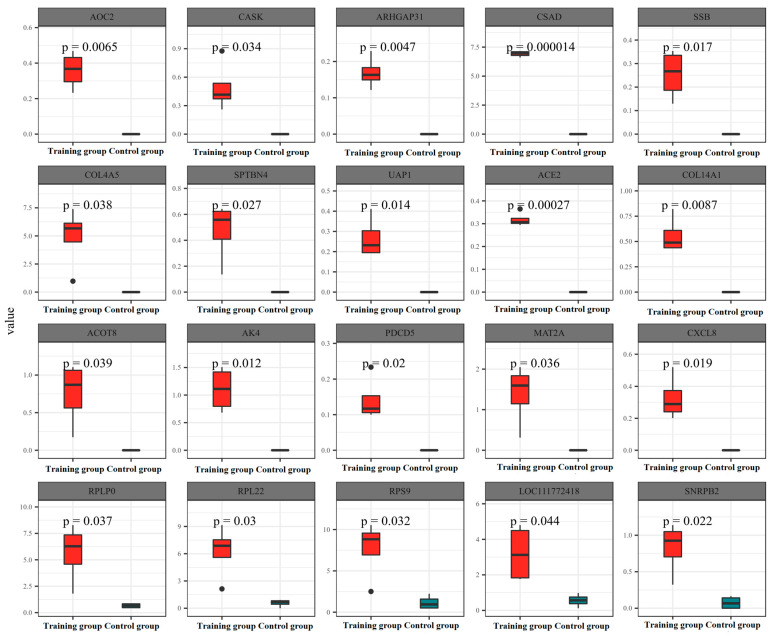
A significant difference analysis performed on the top 20 proteins with different expression levels. Statistical analysis was conducted using a double-tailed *t*-test, and a *p*-value < 0.05 was considered statistically significant.

In an effort to delve deeper into the effects of training on exosomal content, we employed proteomics to analyze the protein expression within the exosomes. Our findings demonstrated that training notably increased the expression of 484 proteins and decreased the expression of 234 proteins (Figure 2A). The majority of these proteins were predominantly localized in the cytoplasm and nucleus (Figure 2B). Further KOG analysis revealed that the principal functional alterations in these proteins pertained to signal transduction processes (Figure 2C). Further significance analysis was conducted on the differentially expressed proteins, showing the top 20 proteins with significant differences (Figure 3).

### 3.3. Training Significantly Altered Metabolome of Plasma Exosomes in the Yili Horse

**Figure 4:**
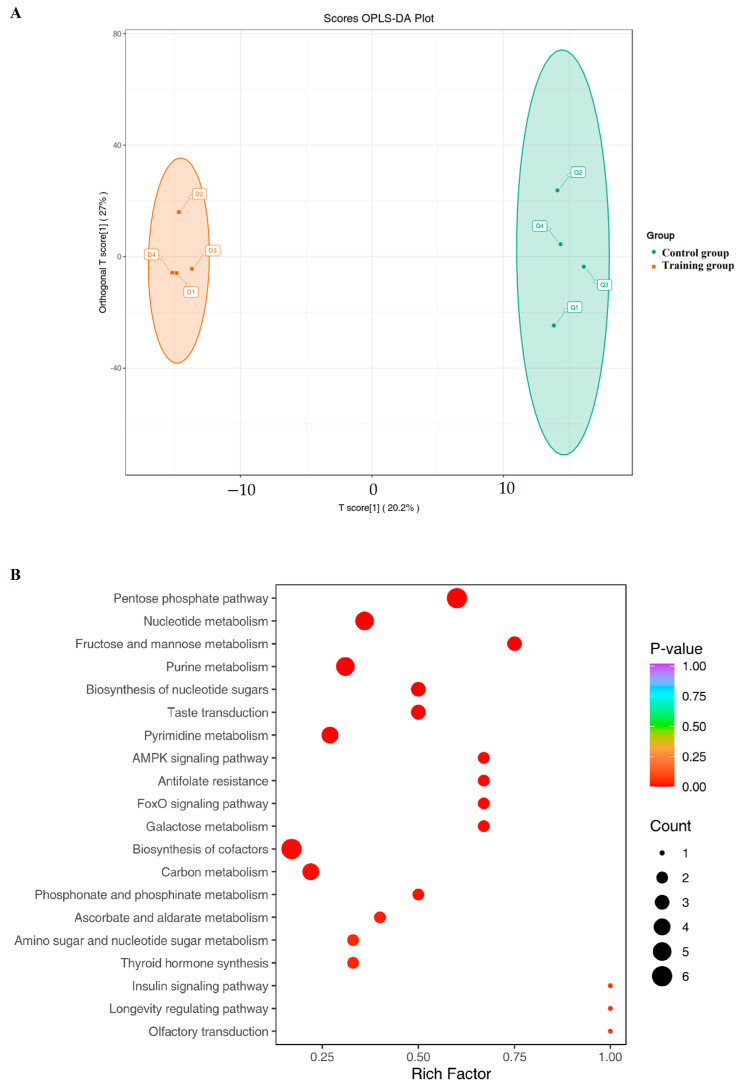
(**A**) OPLS-DA score plot. Note: The horizontal axis represents the predictive principal component; the horizontal axis direction indicates the difference between groups. The vertical axis represents the orthogonal principal component; the vertical axis direction indicates the difference within the group. The percentage represents the explanation rate of this component for the data set. Each point in the figure represents a sample; samples from the same group are represented by the same color. (**B**) KEGG enrichment plot of differential metabolites. The horizontal axis represents the Rich Factor corresponding to each pathway, the vertical axis is the pathway name (sorted by *p*-value), the color of the point reflects the size of the *p*-value; the redder it is, the more significant the enrichment. The size of the point represents the number of differential metabolites enriched.

**Figure 5:**
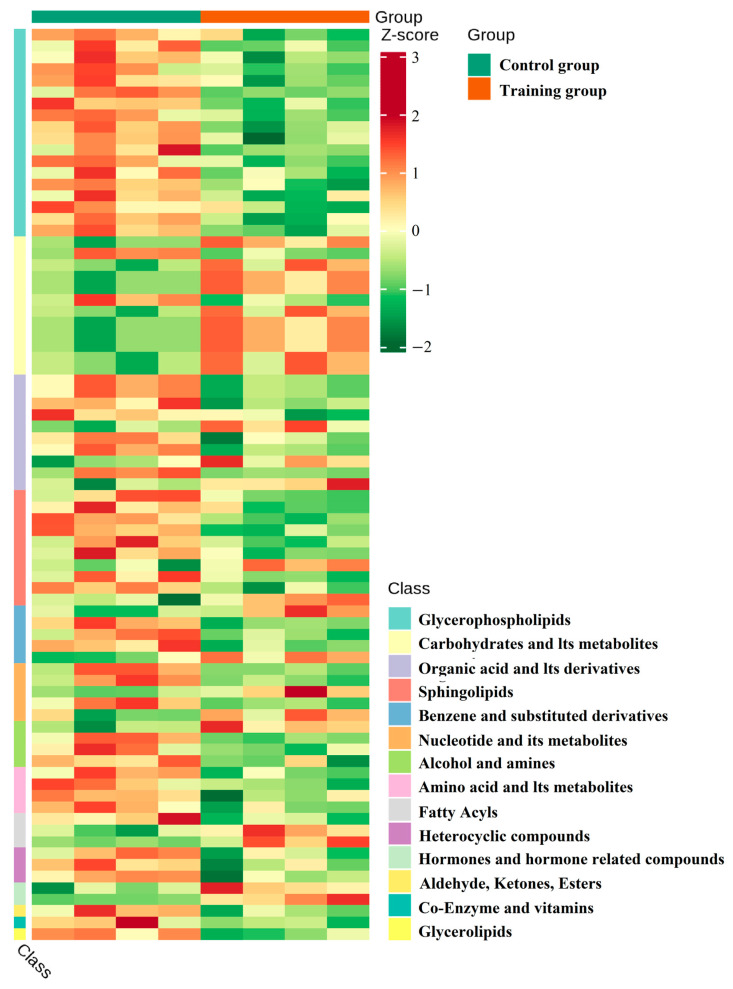
Cluster heatmap of differential metabolites: horizontally for sample names and vertically for differential metabolite information. Different colors represent different values obtained after normalization of different relative contents (red represents high content, while green represents low content). Among them, heatmap class refers to the heatmap by substance classification; class is the primary classification of substances.

**Figure 6:**
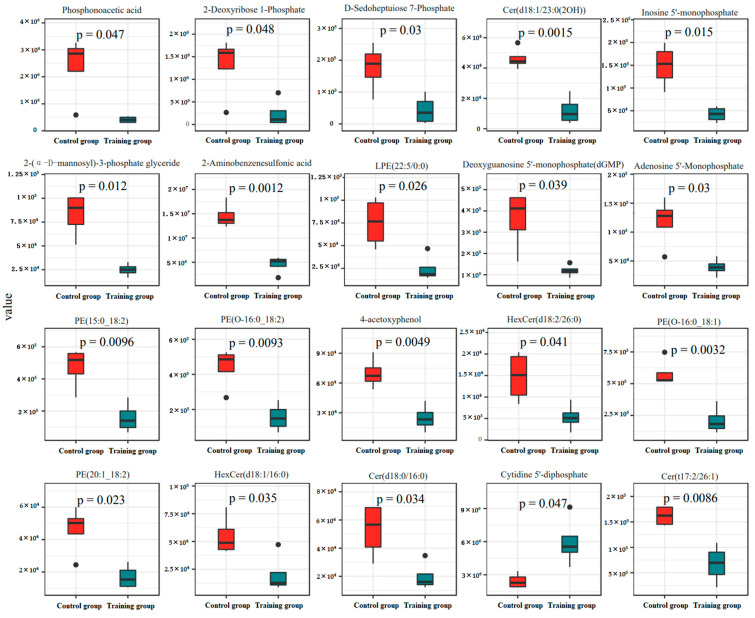
A significant difference analysis performed on the top 20 metabolites with different expression levels. Statistical analysis was conducted using a double-tailed *t*-test, and a *p*-value < 0.05 was considered statistically significant.

After preliminarily determining the impact of training on the metabolic pathways of exosomes, we used LC-MS/MS to detect and compare metabolites. The data showed that training significantly changed the composition of metabolites (Figure 4A). KEGG analysis showed a significant increase in metabolites related to the pentose phosphate pathway, nucleotide metabolism, and fructose and mannose metabolism in the training group (Figure 4B), along with reduced carbohydrates and their metabolites while increasing glycerol phospholipid and organic acids and their derivatives (Figure 5). Further significance analysis was conducted on the differentially metabolites, showing the top 20 proteins with significant differences (Figure 6).

### 3.4. Combined Analysis of the Effects of Training on Yili Horse Plasma Exosome Protein and Metabolites

**Figure 7:**
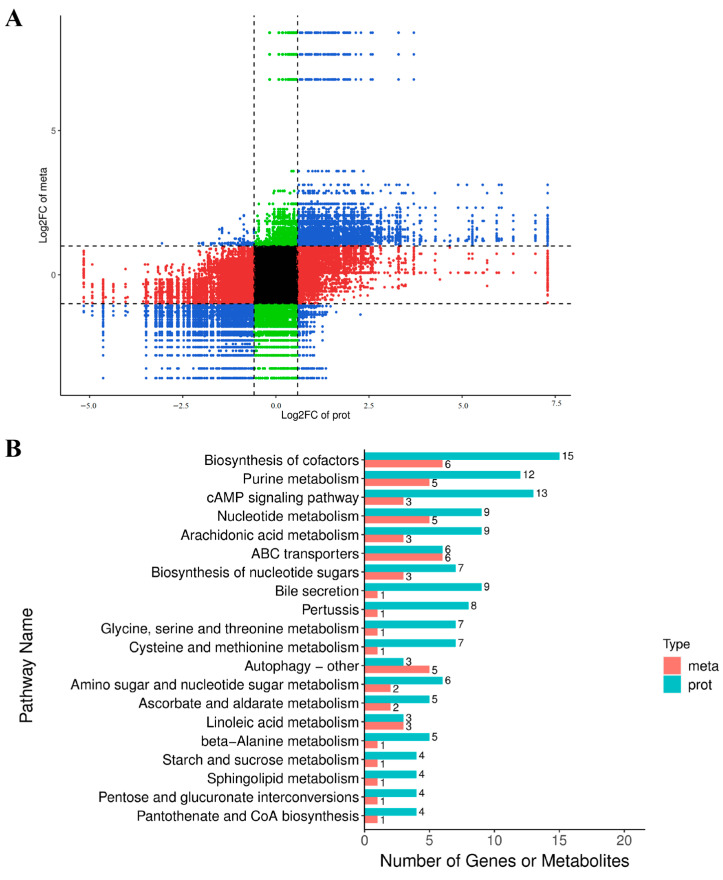
(**A**) Combined analysis of nine quadrant charts in each differential group selects the correlation parts that meet the Pearson correlation coefficient absolute value greater than 0.8 and *p*-value < 0.05, where each point represents a pair of correlation relationships. The horizontal axis represents the Log2FC of the proteins, and the vertical axis represents the Log2FC of the metabolites. Divide into 1–9 quadrants using black dashed lines from left to right and from top to bottom, where proteins and metabolites in quadrant 5 are not differentially expressed. The proteins in quadrants 3 and 7 have a positive correlation with metabolites, and the expression changes in metabolites may be positively regulated by proteins. The proteins in quadrants 1 and 9 have a non-consistent regulatory trend with metabolites, and the expression changes in metabolites may be negatively regulated by proteins. The expression of metabolites in quadrants 2, 4, 6, and 8 remains unchanged, and proteins are upregulated or downregulated, or proteins expression remains unchanged, while metabolites are upregulated or downregulated. (**B**) Based on the KEGG enrichment analysis results of differential proteins and metabolites, analyze the KEGG pathway enriched by both omics, and draw a bar chart. The bar chart shows the number of differentially expressed proteins and metabolites enriched in a certain pathway and only displays the top 20 pathways with the total number of differentially expressed proteins and metabolites.

In order to explore the trend of differential expression of proteins and metabolites in the plasma exosomes of Ili horses after training, we conducted a combined analysis of proteomic and metabolomic data. After a joint analysis of protein and metabolite expression differences in two groups of plasma exosomes, research found that training has similar effects on the expression of proteins and metabolites in *Yili* horse plasma (Figure 7A). Among them, the total number of differences in proteins and metabolites related to the biosynthesis of cofactors, purine metabolism, and the cAMP signaling pathway was the highest (Figure 7B).

## 4. Discussion

Exercise is increasingly recognized as a non-pharmacological intervention for disease management [38], and equine-assisted interventions have been shown to ameliorate certain health conditions [39]. Consequently, promoting equine sports has positive social implications, and objectively assessing the athletic capabilities of horses contributes positively to this field. This study seeks to identify potential biomarkers for evaluating the athletic performance of *Yili* horses by comparing protein expression and metabolite changes in plasma exosomes between trained and untrained horses, thereby analyzing the impact of training on these parameters.

Several studies have linked the health benefits of exercise to exosomes, with exercise improving vascular formation in type 2 diabetes through exosomal mechanisms [40]. Our electron microscopy images indicate that while exercise training does not significantly alter the size of exosomes in *Yili* horses, it does significantly change their plasma exosomal protein expression and metabolite composition.

The development of proteomics and metabolomics technologies has simplified the detection of proteomes and metabolomes [41]. Proteomic and metabolomic analyses have identified metabolic abnormalities in equine follicles matured both in vitro and in vivo [42], which can help optimize breeding strategies. It has been found that different types of exercise lead to significant differences in the proteomic and metabolomic profiles of athletes’ blood [43]. Our study reveals that exercise induces significant changes in the protein expression and metabolites of equine plasma exosomes, with the most numerous differences in proteins associated with the cytoplasm and nucleus, consistent with miRNA changes, suggesting that miRNAs regulate the expression of related proteins. There is a significant difference in the metabolic products of exosomes between the control and trained groups, with a notable reduction in carbohydrates and their metabolites in the trained group, presumably due to the consumption of these substances during exercise training. The pentose phosphate pathway, nucleotide metabolism, and fructose and mannose metabolism are the metabolic pathways with the most significant differences, suggesting that training induces changes in cellular gene expression in *Yili* horses, leading to differences in protein expression and thus altering metabolic capacity, making these metabolism-related metabolites the most significantly altered.

This study showed that training increased the expression of MAT2A (methylene adenosyl transferase II alpha) protein in plasma exosomes of *Yili* horses, which is consistent with the conclusion that exercise upregulation can enhance skeletal muscle MAT2A [44]. After training, the expression of CASK (calmodulin-dependent serine protein kinase) secreted by monocytes and M2 macrophages through exosomes in plasma exosomes increased [45]. This is associated with bone weight and toughness indicators [46], and the upregulated ARHGAP31 (Rho GTPase-activating protein 31) is a growth performance-related gene [47]. SPTBN4 (Spectrin, beta, non-erythrocytic 4) is associated with muscle tone [48], while COL4A5 (Collagen, Type IV, Alpha 5) and COL14A1 (Collagen, Type XIV, Alpha 1) are associated with collagen biosynthesis [49], suggesting that training increases the protein in plasma exosomes that promotes muscle function.

Exercise can regulate the expression of CXCL8 (Interleukin-8) [50,51], which is consistent with the data in this study and may be related to the maintenance and repair of tendons by CXCL8 [52]. This is consistent with the function of RPLP0 (Ribosomal Protein Lateral Stalk Subunit P0) in maintaining the homeostasis of healthy tendons [53] and promoting tissue healing after injury [54], which may explain the increase in CXCL8 and RPLP0 proteins in plasma exosomes of *Yili* horses after training.

AK4 (Adenosine kinase 4) promotes metabolic reprogramming and transfer [55] and cell proliferation [56], regulates purine metabolites [57], and overexpression of CSAD (Cysteine sulfinic acid decarboxylase) can regulate fatty acid metabolism and improve mitochondrial damage in vitro and in vivo [58]. Elevated expression of ACOT8 (Acyl CoA hioesterase 8) is associated with improved lipid metabolism [59,60], suggesting that training alters the metabolic regulatory function of plasma exosome proteins in *Yili* horses.

UAP1 (UDP-N-acetylglucosamine pyrophosphate 1) can regulate metabolism and innate immunity [48], RPL22 (ribosomal protein L22) regulates B cell [61] and T cell development [62], and overexpression of PDCD5 (programmed cell death 5) can induce regulation of CD8+T cells [63], suggesting that training alters the immune regulatory function of plasma exosomes in Ili horses.

Unlike the insignificant trend in protein expression, there was a significant difference in the metabolic products of the two groups of extracellular vesicles, and the top 20 metabolites were all elevated in the training group. Among them, phosphatic acid has antiviral effects [64]. Previous studies have found that training increases the concentration of plasma lysophosphatidyl ethanolamine [65]. The data from this study also shows this change in plasma exosomes. Furthermore, the increase in phosphatidylethanolamine levels may be related to the increase in lysophosphatidyl ethanolamine levels [66]. HexCr may be involved in mediating apoptosis, necrosis, and inflammation levels [67], which may be one of the potential factors leading to patient fatigue, chronic pain, and cognitive difficulties [68], speculated to be related to injury after exercise.

Multiple factors can affect a horse’s athletic ability [69], and genetic characteristics can predict this [70]. Systematic training can also alter protein expression, thereby affecting its athletic ability [71]. Therefore, by analyzing the impact of training on horse plasma exosomes, it is possible to provide objective indicators for evaluating horse sports ability and provide reference for the training and breeding of *Yili* horses. Studies have shown that moderate exercise enhances the release and function of endothelial progenitor cell exosomes [72], suggesting that training may also affect the overall level of plasma exosomes in *Yili* horses, warranting further investigation.

Studies have indicated that exercise-induced changes in plasma proteins are related to gender [73], and whether exercise-induced changes in exosomal proteins are also gender-related merits further study. Purebred and non-purebred Jeju horses exhibit different plasma metabolites after exercise [74], and we believe it is necessary to further compare the plasma metabolites of purebred and crossbred *Yili* horses post-exercise to obtain more biological reference indicators.

## 5. Conclusions

Our research finds that training significantly alters the protein expression and metabolomics of plasma exosomes in *Yili* horses, with these changes primarily concentrated on promoting muscle function and repair, regulating metabolism and immunity, and reducing carbohydrates and their metabolites. This finding provides new insights into the training and assessment of athletic abilities in *Yili* horses, but further research is still needed.

## Data Availability

All data that support the results of this study are available from the corresponding author on reasonable request.

## Abbreviations

SEM	Scanning Electron Microscopy
KOG	Cluster of Orthologous Groups of proteins
GO	Gene Ontology
KEGG	Kyoto Encyclopedia of Genes and Genomes
LC-MS/MS	Liquid Chromatography–Tandem Mass Spectrometry
Ns	Not Significant
EDTA-K2	Dipotassium Ethylenediaminetetraacetate
BCA	Bicinchoninic Acid Assay
DTT	Dithiothreitol
IAM	Iodoacetamide
timsTOF Pro2	Trapped Ion Mobility Spectrometry Time-of-Flight Mass Spectrometer Pro 2
OPLS-DA	Orthogonal Partial Least Squares Discriminant Analysis
MAT2A	Methylene adenosyl transferase II alpha
CASK	Calmodulin-dependent serine protein kinase
ARHGAP31	Rho GTPase-activating protein 31
SPTBN4	Spectrin, beta, non-erythrocytic 4)
COL4A5	Collagen, Type IV, Alpha 5
COL14A1	Collagen, Type XIV, Alpha 1
CXCL8	Interleukin-8
RPLP0	Ribosomal Protein Lateral Stalk Subunit P0
AK4	Adenosine kinase 4
CSAD	Cysteine sulfinic acid decarboxylase
ACOT8	Acyl CoA hioesterase 8
UAP1	UDP-N-acetylglucosamine pyrophosphate 1
RPL22	Ribosomal protein L22
